# A simplified model for prophylactic transarterial chemoembolization after resection for patients with hepatocellular carcinoma

**DOI:** 10.1371/journal.pone.0276627

**Published:** 2022-10-31

**Authors:** Nanping Lin, Lei Wang, Qizhen Huang, Weiping Zhou, Xiaolong Liu, Jingfeng Liu

**Affiliations:** 1 Department of Hepatopancreatobiliary Surgery, The First Affiliated Hospital of Fujian Medical University, Fuzhou, China; 2 Fujian Provincial Cancer Hospital, Fuzhou, China; 3 Department of Hepatopancreatobiliary Surgery, Mengchao Hepatobiliary Hospital of Fujian Medical University, Fuzhou, China; 4 The Third Department of Hepatic Surgery, Eastern Hepatobiliary Surgery Hospital, Secondary Military Medical University, Shanghai, China; 5 The United Innovation of Mengchao Hepatobiliary Technology Key Laboratory of Fujian Province, Mengchao Hepatobiliary Hospital of Fujian Medical University, Fuzhou, China; Medizinische Fakultat der RWTH Aachen, GERMANY

## Abstract

**Background:**

Prophylactic transarterial chemoembolization (p-TACE) is frequently conducted for patients with hepatocellular carcinoma (HCC) in China, but the question of who could benefit from it remains controversial. Hence, we wanted to establish a nomogram model to identify patients eligible for p-TACE.

**Methods:**

Data from HCC patients receiving R0 resection with or without p-TACE between January 2013 and December 2014 were identified, using primary liver cancer big data, to establish a nomogram model to predict overall survival (OS). Based on the model, Patients receiving R0 resection between January 2015 and December 2015 were divided into three subgroups, and survival curves were constructed using the Kaplan–Meier method and analyzed by the log-rank test among patients in each subgroup.

**Results:**

A nomogram integrating the neutrophil to lymphocyte ratio, AFP, tumor diameter, and microvascular invasion was developed to predict the OS of patients with HCC receiving R0 resection, and significant differences were observed in the median OS of the subgroups of low-risk (≤20), intermediate-risk (20~120), and high-risk (>120) identified by the current model. This model showed good calibration and discriminatory power in the validation cohort and the external cohort (c-index of 0.669 and 0.676, respectively). In the external cohort, the Kaplan–Meier curves showed that p-TACE could only significantly prolong the median OS of high-risk patients (25.6 vs. 33.7 months, P<0.05), but no differences were observed in any subgroups stratified by the current staging systems (all P>0.05).

**Conclusion:**

This readily available nomogram model could help guide decisions about p-TACE, but it needs further validation.

## Introduction

Hepatocellular carcinoma is the sixth most common cancer globally, and approximately 850,000 patients are newly diagnosed with HCC every year [1]. Surgical resection is still the preferred strategy worldwide, although great progress has been made in the fields of transplantation, radiofrequency ablation (RFA), and stereotactic body radiotherapy (SBRT) [2]. Nonetheless, the long-term prognosis of patients receiving surgical resection remains far from satisfactory, mainly owing to the high incidence of intrahepatic recurrence. Transarterial chemoembolization (TACE) have been applied in recent decades to prevent recurrences and improve the prognosis [3], but unfortunately, neither has been widely confirmed nor recommended by current guidelines for that uses [4, 5].

Among the adjuvant strategies, prophylactic TACE (p-TACE) has become dominant in China [6], although the underlying mechanism of p-TACE remains controversial [6, 7]. p-TACE has been verified to benefit patients who are “high risk” by numerous studies and meta-analyses [8–10], but agreements on the definition of “high risk” are far from being reached. In addition, selecting patients to receive p-TACE according to the so-called “high-risk factors” is generally impractical [11]. Hence, an accurate and user-friendly model for patients receiving surgical resection to identify the candidates that will benefit the most from p-TACE is urgently needed.

In the current study, data of HCC patients receiving R0 resection were extracted from primary liver cancer big data (PLCBD) in China to establish and validate a selection model for patients who should receive p-TACE following R0 resection.

## Materials and methods

### Patients

This study was approved by our institution’s Ethics Committee (No. 2019_039_01) and performed according to the Declaration of Helsinki. Due to the retrospective nature of the study, informed consent was waived.

Patients receiving surgical resection but no p-TACE from January 2013 to December 2014 were identified to establish a prediction model, which was randomly divided into training and validation cohorts. Patients treated from January 2015 to December 2015 were used to verify whether the current model could identify the potential beneficiaries of p-TACE, which was used as the external cohort.

Patients who underwent R0 resection and were diagnosed with HCC by pathology were eligible for this study. Patients were excluded from this study if they met the following criteria: 1) resection for relapsed HCC, 2) distant metastasis, 3) macrovascular or bile duct tumor thrombus, 4) other malignant cancers, 5) death within four weeks following surgery, and 6) preoperative treatment or any adjuvant treatments, 7) tumor residue found by p-TACE angiography, or 8) incomplete data.

The data of patients receiving surgical resection were extracted from PLCBD by an IT engineer, and they were independently verified by the researchers: age, sex, HBV infection, serum AFP level, Child–Pugh grading, cirrhosis, intraoperative transfusion, tumor number, tumor diameter, differentiation grading, capsule, presence of tumor satellites, and outcomes.

### Interventions and definitions

Before hepatectomy and TACE, all laboratory values, including albumin, bilirubin, neutrophil, and lymphocyte counts, AFP levels, and viral tests (HBsAg, HBeAg, and HBV-DNA); routine imaging, including abdominal ultrasound, dynamic contrast-enhanced computed tomography (CT) and/or magnetic resonance imaging (MRI); and dynamic changes in liver function evaluated by the Child–Pugh score or the indocyanine green clearance rate at 15 minutes (ICG-15); were conducted.

Hepatectomy was generally performed openly or laparoscopically via the anterior approach. Major hepatectomy was defined as the resection of Couinaud segments ≥3, applied only to those with Child–Pugh class A and ICG-15 ≤ 15%.

Briefly, p-TACE was conducted based on the consensus of Chinese experts [12], performed within four to eight weeks after hepatectomy. Under digital subtraction angiography (DSA), an 5-F catheter or microcatheter was selectively inserted into the predesigned hepatic artery, and then an emulsion of lipiodol (2–5 mL) was infused instantly after a slow injection of cisplatin (10–30 mg), doxorubicin hydrochloride (10 mg) and pharmorubicin (20–40 mg).

Adverse events (AEs) related to surgery and TACE were subjected to the Clavien–Dindo classification [13] and were extracted from the medical records, and grade III and above AEs were defined as severe AEs.

### Follow-up and endpoints

Patients were followed up periodically based on the Chinese guidelines for treating primary liver cancer [11]. Generally, routine items of serum AFP and abdominal ultrasound were examined at three-month intervals after resection. Dynamic enhanced CT/MRI was performed when a recurrence was clinically suspected. Further intervention was immediately started when a recurrence was confirmed, including re-resection, RFA, TACE, and SBRT.

The primary endpoint in this study was overall survival (OS), which was calculated from the date of resection to either the date of death or the last follow-up (October 2018).

### Clinical and pathological variables

Generally, all the variables potentially associated with the OS of HCC were reclassified based on previous studies [14, 15]. Specifically, the tumor number was categorized as single vs. multiple according to the National Comprehensive Cancer Network (NCCN) guidelines on HCC [4], and differentiation was categorized as I/II vs. III/IV according to the Edmondson-Steiner grading system [16]. Microvascular invasion (MVI) detected by pathologist after surgery. Of note, regardless of the status of HBsAg and HBV-DNA, HBV infection was defined as a history of HBV infection. Blood transfusion included intraoperative transfusion of red blood cells and plasma, which was extracted from the anesthesia records. Age was categorized as<50 years vs. ≥50 years as previously reported [17], and serum AFP levels were categorized as <400 ng/ml vs. ≥400 ng/ml using the preferred cutoff value [18, 19]. The neutrophil to lymphocyte ratio (NLR) was calculated initially and then dichotomized as low or high. The albumin-bilirubin score (ALBI) was calculated based on the following formula: (-0.085× (albumin g/L) + 0.66 × log (bilirubin μmol/L)) and graded as 1 (score ≤-2.60), 2 (score -2.6 to -1.39), or 3 (score>-1.39) based on a previous report [20].

### Statistics

Variables were compared using the t-test, chi-square test, or Fisher’s exact test (two-tailed). The variable NLR was dichotomized for OS using the optimal cutoff values determined by the “surv_cutpoint” function of the “survminer” package [21]. Variables associated with OS were identified using the univariate Cox model, and those with P< 0.05 were then used to conduct multivariate Cox regression through backward selection to identify independent prognostic factors.

A nomogram was developed based on the Cox multivariate analysis of OS through the “rms” package in R. The performance of the new nomogram was evaluated using the concordant index (C-index) and calibration with 1000 bootstrap samples and then was compared with current staging systems of the Barcelona Clinic Liver Cancer (BCLC) [5], and China Liver Cancer (CNLC) [11] using the package “rcorrp.cens”.

In the external cohort, survival curves were depicted using the Kaplan–Meier method between the groups of p-TACE and non-TACE in each subgroup and compared using the log-rank test with 95% confidence intervals (CIs).

All analyses were conducted via R project version 3.6.3 (http://www.r-project.org/), and P <0.05 with two tails was considered statistically significant.

## Results

### Patients’ characteristics and prognosis

Initially, 744 patients receiving R0 resection from January 2013 to December 2014 were divided into training and validation cohorts. The basic characteristics of the training and validation cohorts are shown in Table 1. The optimal cutoff value for NLR was 2.46 for OS (S1 Fig); hence, an NLR score <2.46 was defined as low and ≥2.46 as high in this study.

**Table 1:** The basic characteristic of the training cohort and validation cohort.

Characteristic	Primary cohort		P-value
	Training cohort (n = 372)	Internal validation cohort (n = 372)	
**Age**			0.883
≤50 year	167 (44.9%)	170 (45.7%)	
>50 year	205 (55.1%)	202 (54.3%)	
**Sex**			0.018
Female	44 (11.8%)	68 (18.3%)	
Male	328 (88.2%)	304 (81.7%)	
**NLR**			0.156
Low level	264 (71.0%)	245 (65.9%)	
High level	108 (29.0%)	127 (34.1%)	
**ALBI**			0.367
1 grade	163 (43.8%)	157 (42.2%)	
2 grade	196 (52.7%)	194 (52.2%)	
3 grade	13 (3.5%)	21 (5.6%)	
**HBV** ^*^			0.440
No	51 (13.7%)	43 (11.6%)	
Yes	321 (86.3%)	329 (88.4%)	
**Cirrhosis**			1.000
No	113 (30.4%)	114 (30.6%)	
Yes	259 (69.6%)	258 (69.4%)	
**AFP**			0.636
<400ng/ml	251 (67.5%)	258 (69.4%)	
≥400ng/ml	121 (32.5%)	114 (30.6%)	
**Tumor number**			0.407
Single	322 (86.6%)	313 (84.1%)	
Multiple	50 (13.4%)	59 (15.9%)	
**Tumor diameter**			0.187
Mean (SD)	5.66(3.80)	6.05(4.28)	
**Capsule**			0.651
Complete	60 (16.1%)	55 (14.8%)	
Incomplete	248 (66.7%)	255 (68.5%)	
Missing	64 (17.2%)	62 (16.7%)	
**Presence of tumor satellites**			0.704
No	231 (62.1%)	237 (63.7%)	
Yes	141 (37.9%)	135 (36.3%)	
**Differentiation**			0.433
Ⅰ/Ⅱgrade	36 (9.7%)	40 (10.8%)	
Ⅲgrade	303 (81.5%)	308 (82.8%)	
Ⅳgrade	33 (8.9%)	24 (6.5%)	
MVI*			0.875
No	254 (68.3%)	251 (67.5%)	
Yes	118 (31.7%)	121 (32.5%)	

NLR, neutrophils to lymphocyte ratio; ALBI, albumin- bilirubin grade; HBV, hepatitis B virus; AFP, alpha-fetoprotein; MVI, microvascular invasion.

### Independent prognostics factors for OS

NLR, ALBI, AFP, tumor number, tumor diameter, capsule, presence of tumor satellites, tumor differentiation, and MVI were identified as prognostic factors for OS (all P<0.05, Table 2) using univariate analysis, but only NLR (HR = 1.80, 95%CI = 1.27–2.56), AFP (HR = 1.94, 95%CI = 1.38–2.75), tumor diameter (HR = 1.09, 95%CI = 1.04–1.13), and MVI (HR = 1.48, 95%CI = 1.01–2.17) remained independent risk factors for OS (all P<0.05, Table 2) in the multivariate Cox model.

**Table 2:** Uni- and multi-variate Cox regression analysis for overall survival in the training cohort.

Characteristic	Univariate		Multivariate	
	HR (95CI)	*p*-Value	HR (95CI)	*p*-Value
**Age**		0.81		
≤50 years	Ref.			
>50years	1.04(0.75–1.45)			
**Sex**		0.967		
Female	Ref.			
Male	0.99(0.60–1.64)			
**NLR**		<0.001		0.001
Low level	Ref.			
High level	2.11(1.5–2.95)		1.80(1.27–2.56)	
**ALBI**				
1 grade	Ref.			
2 grade	1.2(0.85–1.69)	0.299	0.90(0.63–1.29)	0.577
3 grade	3.83(1.88–7.78)	<0.001	0.99(0.41–2.39)	0.981
**HBV**		0.887		
No	Ref.			
Yes	1.04(0.62–1.73)			
**Cirrhosis**		0.143		
No	Ref.			
Yes	0.77(0.55–1.09)			
**AFP**		<0.001		<0.001
<400ng/ml	Ref.			
≥400ng/ml	2.30(1.65–3.20)		1.94(1.38–2.75)	
**Transfusion**		0.071		
No	Ref.			
Yes	1.81(0.95–3.45)			
**Tumor number**		0.005		0.777
Single	Ref.			
Multiply	1.32(1.09–1.60)		0.97(0.76–1.23)	
**Tumor diameter**		<0.001		<0.001
<5cm	Ref.			
≥5cm	1.12(1.08–1.16)		1.09(1.04–1.13)	
**Capsule**				
Complete	Ref.			
Incomplete	1.34(0.81–2.22)	0.253		
Missing	1.53(0.84–2.79)	0.160		
**Satellite**		0.006		
No	Ref.			
Yes	1.60(1.15–2.23)			
**Differentiation**				
Ⅰ/Ⅱgrade	Ref.			
Ⅲgrade	2.09(1.02–4.28)	0.044	1.39(0.67–2.90)	0.332
Ⅳgrade	2.52(1.05–6.03)	0.038	1.13(0.46–2.79)	0.122
**MVI**		<0.001		0.043
No	Ref.			
Yes	2.01(1.44–2.80)		1.48(1.01–2.17)	

NLR, neutrophils to lymphocyte ratio; ALBI, albumin- bilirubin grade; HBV, hepatitis B virus; AFP, alpha-fetoprotein; MVI, microvascular invasion.

### Development and validation of the selection model

**Fig 1:**
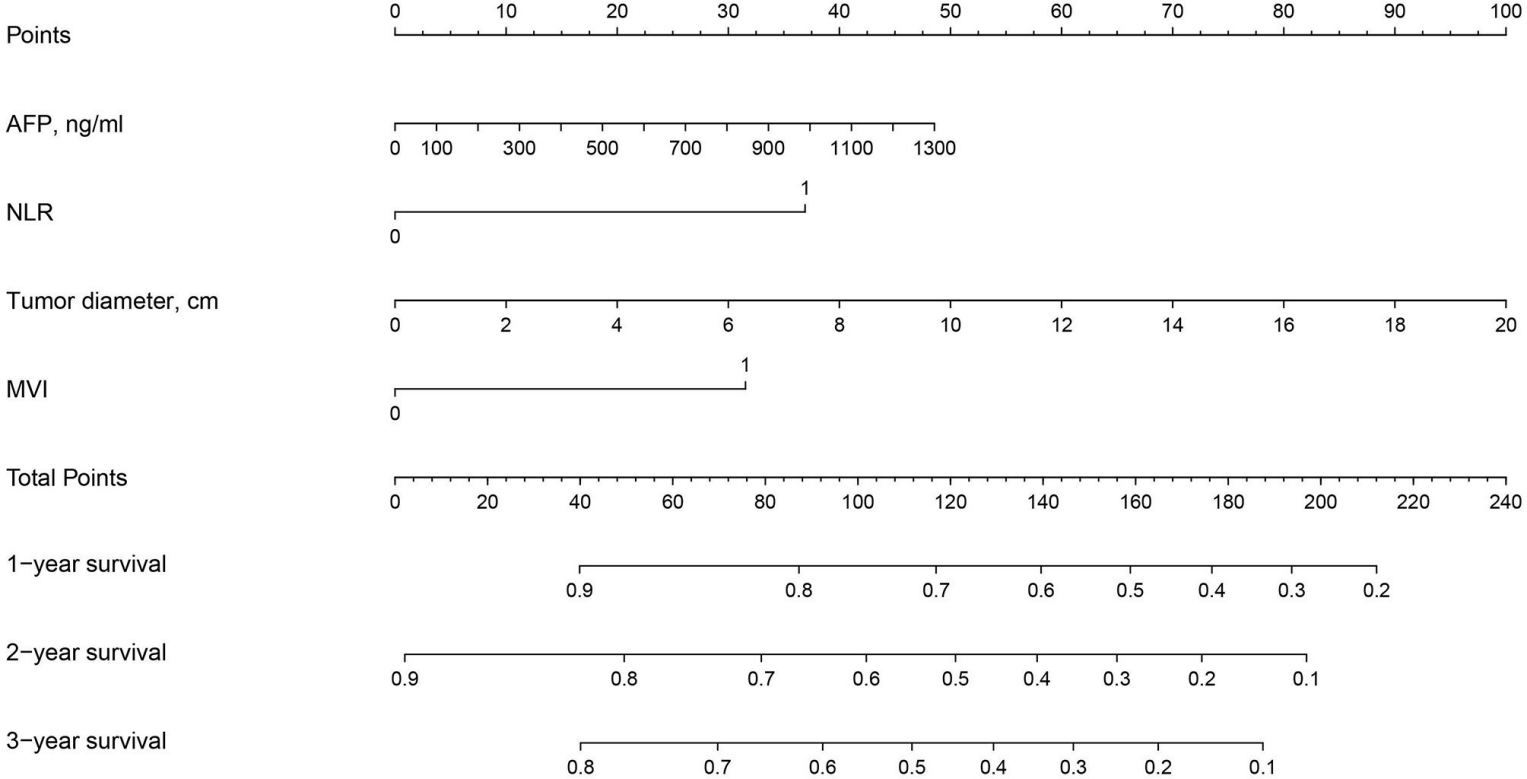
Nomogram for predicting overall survival of patients receiving R0 resection.

**Fig 2:**
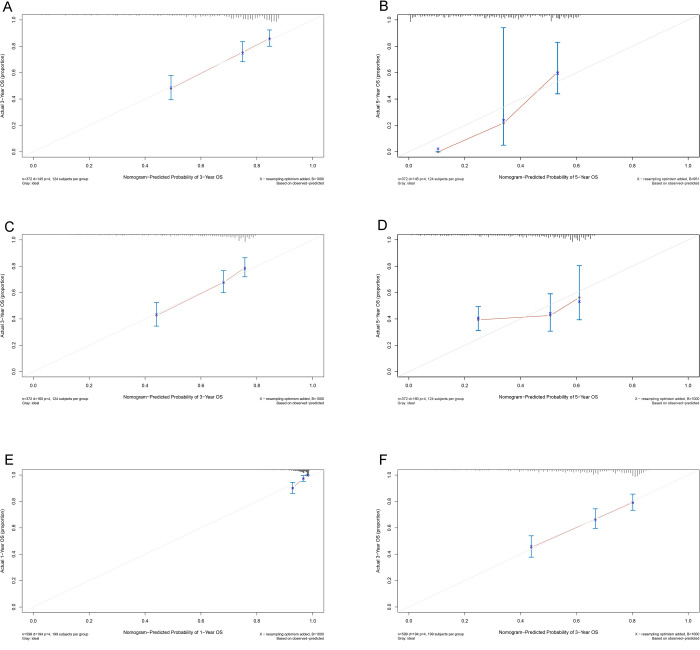
The calibration curve for predicting overall survival by the nomogram at 3 year and 5 years in training cohort (A, B), validation cohort (C, D) and 1 year and 3 years in external cohort (E, F).

A nomogram consisting of all the independent risk factors for OS was established, as shown in Fig 1. The C-index for the OS prediction was 0.703 (95% CI, 0.659–0.747), and the calibration curves for the probabilities of OS at 3 and 5 years after hepatectomy exhibited optimal agreement between the prediction by the nomogram and the actual observation (Fig 2A and 2B).

In the validation cohort, the C-index for OS prediction was 0.669 (95% CI, 0.628–0.710). The calibration plots showed satisfactory consistency for the probabilities of OS after hepatectomy between those predicted by the nomogram and the observed probabilities for 3- and 5-year OS (Fig 2C and 2D).

### Comparison between the current nomogram model and other staging models in the training cohort

The discriminatory power of the current nomogram model and other widely used staging models, including the BCLC, and CNLC staging systems, were compared using the C-index in the training cohort. The current nomogram model has the highest discriminatory power in predicting the OS of patients receiving R0 resection, and its C-index was 0.703 (95% CI: 0.659–0.747), which was higher than the staging systems of, BCLC (C-index, 0.667, 95% CI: 0.620–0.714, P = 0.047), and CNLC (C-index, 0.668, 95% CI: 0.621–0.714, P = 0.046, Table 3).

**Table 3:** Comparison of C-index between the nomogram, BCLC and CNLC models for the prediction of overall survival.

Models	C-index	95%CI	P-value
**Nomogram**	0.703	0.659–0.747	Ref.
**AJCC**	0.683	0.637–0.728	0.179
**BCLC**	0.667	0.620–0.714	0.047
**CNLC**	0.668	0.621–0.714	0.046

AJCC, American Joint of Cancer Committee; BCLC Barcelona Clinic Liver Cancer; CNLC, China Liver Cancer.

### Risk stratification based on the current nomogram model

**Fig 3:**
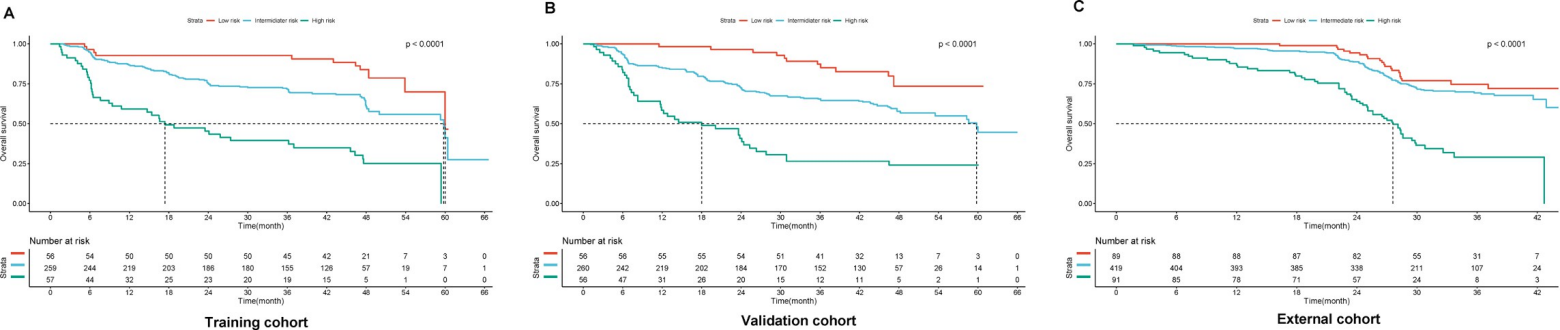
Kaplan-Meier curve of OS for subgroups stratified by the nomogram model in the training cohort (A), validation cohort (B) and external cohort (C).

The median OS in the training and validation cohorts was 34.8 and 34.4 months, respectively. Using 20 and 120 as the cutoff values of the current nomogram model, which corresponded to the 15^th^ and 85^th^ centiles of the score in the derivation cohort, patients in the derivation cohort were divided into three subgroups: low-risk, intermediate-risk, and high-risk, respectively. Patients in the three subgroups exhibited a well-stratified prognosis: low-risk (3-year OS, 80.3%), intermediate-risk (3-year OS, 59.8%), and high-risk (3-year OS, 33.3%) (P<0.001, Fig 3A). Based on the cutoff values selected in the training cohort, good prognostic stratification was observed among all the subgroups in terms of OS in the validation cohort (P<0.001, Fig 3B) and the external cohort (P<0.001, Fig 3C).

### Survival curves for patients receiving p-TACE or not stratified by subgroups according to the current nomogram model

Patients receiving R0 resection from January 2015 to December 2015 were enrolled as the external cohort, including 108 patients (108/599, 18.0%) who received p-TACE. Their basic characteristics are depicted in Table 4. Notably, the proportions of HBV infection, multiple tumors, presence of tumor satellites, and MVI were prominently higher in the TACE group (all P<0.05, Table 4).

**Table 4:** Basic characteristics between TACE and non-TACE in external cohort.

Characteristic	Non-TACE (n = 491)	TACE (n = 108)	P-value
**Age**			
≤50 years	186 (37.9%)	45 (41.7%)	0.534
>50years	305 (62.1%)	63 (58.3%)	
**Sex**			
Female	79 (16.1%)	14 (13.0%)	0.506
Male	412 (83.9%)	94 (87.0%)	
**NLR**			
Low level	356 (72.5%)	72 (66.7%)	0.272
High level	135 (27.5%)	36 (33.3%)	
**ALBI**			
1 grade	197 (40.1%)	49 (45.4%)	0.301
2 grade	269 (54.8%)	51 (47.2%)	
3 grade	25 (5.1%)	8 (7.4%)	
**HBV**			
No	103 (21.0%)	13 (12.0%)	0.046
Yes	388 (79.0%)	95 (88.0%)	
**Cirrhosis**			
No	189 (38.5%)	39 (36.1%)	0.725
Yes	302 (61.5%)	69 (63.9%)	
**AFP**			
<400ng/ml	329 (67.0%)	65 (60.2%)	0.215
≥400ng/ml	162 (33.0%)	43 (39.8%)	
**Tumor number**			
Single	396 (80.7%)	66 (61.1%)	<0.001
Multiply	95 (19.3%)	42 (38.9%)	
**Tumor diameter**			
Mean (SD)	5.89 (3.82)	6.46 (3.91)	0.168
**Capsule**			
Complete	81 (16.5%)	17 (15.7%)	0.598
Incomplete	289 (58.9%)	74 (68.5%)	
Missing	121 (24.6%)	17 (15.7%)	
**Satellite**			
No	246 (50.1%)	34 (31.5%)	<0.001
Yes	245 (49.9%)	74 (68.5%)	
**Differentiation**			
Ⅰ/Ⅱgrade	28 (5.7%)	6 (5.6%)	0.988
Ⅲgrade	433 (88.2%)	95 (88.0%)	
Ⅳgrade	30 (6.1%)	7 (6.5%)	
**MVI**			
No	275 (56.0%)	44 (40.7%)	0.006
Yes	216 (44.0%)	64 (59.3%)	

NLR, neutrophils to lymphocyte ratio; ALBI, albumin- bilirubin grade; HBV, hepatitis B virus; AFP, alpha fetoprotein; MVI, microvascular invasion.

The C-index for OS prediction was 0.676 (95% CI, 0.640–0.714), and the calibration curves for the probability of OS at 1 and 3 years after hepatectomy exhibited satisfactory agreement between the prediction by the nomogram and the actual observation (Fig 2E and 2F).

**Fig 4:**
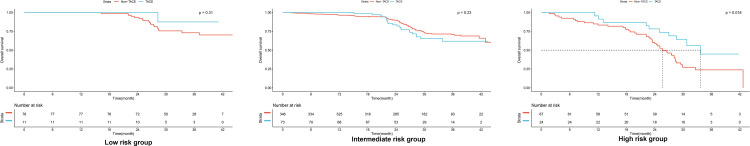
Kaplan-Meier curve for patients receiving p-TACE or not in the external cohort stratified by subgroups according to the nomogram models.

**Fig 5:**
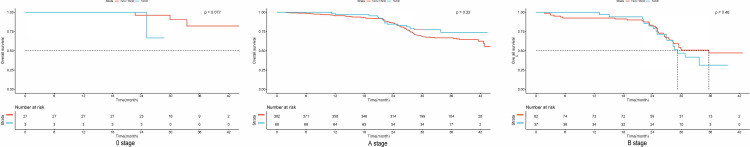
Kaplan-Meier curve for patients receiving p-TACE or not in the external cohort stratified by subgroups according to the BCLC stage.

**Fig 6:**
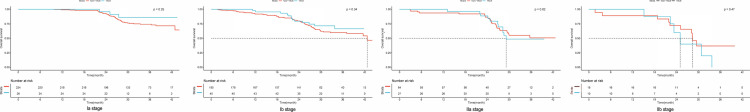
Kaplan-Meier curve for patients receiving p-TACE or not in the external cohort stratified by subgroups according to the CNLC stage.

Based on the current nomogram model and the cutoff values established in the training cohort, patients in the external cohort were divided into three subgroups: 89 patients in the low-risk subgroup, 419 patients in the intermediate-risk subgroup, and 91 patients in the high-risk subgroup. The median OS of patients in the high-risk subgroup receiving p-TACE was significantly longer than that of patients receiving R0 resection only (25.6 vs. 33.7 months, P<0.05, Fig 4), but no difference was observed in any subgroup stratified by the staging systems of BCLC, or CNLC (all P>0.05, Figs 5 and 6). The basic characteristics of high-risk subgroup are depicted in Table 5.

**Table 5:** Basic characteristics between TACE and non-TACE in high risk group of external cohort.

Characteristic	Non-TACE (n = 67)	TACE (n = 24)	P-value
**Age**			
≤50 years	31 (46.3%)	11 (45.8%)	1
>50years	36 (53.7%)	13 (54.2%)	
**Sex**			
Female	19 (28.4%)	3 (12.5%)	0.201
Male	48 (71.6%)	21 (87.5%)	
**NLR**			
Low level	15 (22.4%)	4 (16.7%)	0.765
High level	52 (77.6%)	20 (83.3%)	
**ALBI**			
1 grade	19 (28.4%)	6 (25.0%)	0.904
2 grade	39 (58.2%)	14 (58.3%)	
3 grade	9 (13.4%)	4 (16.7%)	
**HBV**			
No	19 (28.4%)	1 (4.2%)	0.0301
Yes	48 (71.6%)	23 (95.8%)	
**Cirrhosis**			
No	33 (49.3%)	12 (50.0%)	1
Yes	34 (50.7%)	12 (50.0%)	
**AFP**			
<400ng/ml	9 (13.4%)	2 (8.3%)	0.77
≥400ng/ml	58 (86.6%)	22 (91.7%)	
**Tumor number**			
Single	48 (71.6%)	14 (58.3%)	0.344
Multiply	19 (28.4%)	10 (41.7%)	
**Tumor diameter**			
Mean (SD)	10.8(4.82)	11.5 (3.85)	0.501
**Capsule**			
Complete	5 (7.5%)	1 (4.2%)	0.729
Incomplete	39 (58.2%)	19 (79.2%)	
Missing	23 (34.3%)	4 (16.7%)	
**Satellite**			
No	25 (37.3%)	8 (33.3%)	0.92
Yes	42 (62.7%)	16 (66.7%)	
**Differentiation**			
Ⅲgrade	58 (86.6%)	22 (91.7%)	0.77
Ⅳgrade	9 (13.4%)	2 (8.3%)	
**MVI**			
No	12 (17.9%)	9 (37.5%)	0.0945
Yes	55 (82.1%)	15 (62.5%)	

NLR, neutrophils to lymphocyte ratio; ALBI, albumin- bilirubin grade; HBV, hepatitis B virus; AFP, alpha fetoprotein; MVI, microvascular invasion.

## Discussion

In the current study, we developed a nomogram model that had higher discriminatory power than that of the current staging systems. The Kaplan–Meier curves showed that p-TACE could only benefit patients scoring >120 as determined by the nomogram model but not any subgroup stratified by the current staging systems.

p-TACE is frequently conducted with the initial aim of preventing recurrences, but its efficacy remains controversial [22, 23]. The reasons might be as follows: 1) great heterogeneity exists among HCC patients undergoing resection [14], 2) the mechanisms by which p-TACE may prevent recurrence remain unknown [24], and 3) adverse effects, including hepatic dysfunction and immune function impairment, are hard to avoid [25]. Nonetheless, p-TACE has been identified repeatedly as an independent protective factor for OS, which is often considered to be a hard endpoint of studies [3, 24, 25]. Hence, we selected OS as the primary endpoint rather than recurrence or DFS.

The novelty of this study lies in its methodology. Patients with “high-risk” factors, including aggressive tumor characteristics, are recommended to receive p-TACE [11, 12], but the definition of “high-risk” is hard to standardize and normalize [3, 24]. A model incorporating “high-risk” factors is the solution to this difficult problem. In the current study, we selected patients receiving R0 resection without p-TACE to establish a prediction model and then selected an external cohort including patients with or without p-TACE to verify whether the model could guide the application of p-TACE. Different from previous models [26, 27], which selected patients receiving p-TACE to establish models directly, the methodology applied in this study had several advantages: 1) it decreased the interference of p-TACE because systematic reviews and meta-analyses failed to confirm a definite benefit of p-TACE [9, 28]; and 2) it complied with the clinical treatment process completely because the surgical margin and postoperative pathology were the determinant factors of TACE.

The major advantage of the current nomogram model is its ready availability. AFP is an important part of the diagnosis of HCC, and elevated preoperative AFP levels often indicate a potential recurrence and a worse prognosis [18, 19, 29]. Diameter is an essential element of the tumor that can be easily measured [30]. MVI has been well studied in the last decade and is often deemed the origin of intrahepatic recurrence [23, 31]. More importantly, the NLR is considered to be a new biomarker of recurrence and prognosis [32]; it is also readily available using preoperative routine blood examinations and represents the systematic immune status. In this study, the current model not only showed optimal performance for predicting OS among patients receiving R0 resection but it also exhibited good stratification among patients receiving p-TACE, indicating that the current model is robust.

To the best of our knowledge, p-TACE is rarely referred to in the current guidelines, especially in Western countries [4, 5]. p-TACE is recommended for patients with “high-risk” factors in the new CNLC [11], but the definition of “high risk” is not clear, which makes it unfeasible in the clinic. Based on the current model, patients were divided into three subgroups, and a survival benefit from p-TACE was observed only in the subgroup of patients with scores >120 according to the current model, which indicated that the current model could guide the application of p-TACE.

There are several limitations of this study. First, considering the differences in HCC between the east and the west [1], the conclusion needs further validation in the west. Second, variates of AFP is not always measured in Western countries according to current guidelines [2], which suggests that the conclusion may not be directly applicable to patients in Western countries. Third, recall bias and selection bias are hard to avoid in a retrospective study. Fourth, various treatments were immediately adopted once a recurrence was confirmed, which could influence the OS of the patients. Last but not least, with limited patients, the conclusion in low-risk group needed further validation, even the enrolled patients were higher in high-risk group, a further study is warrant to variated the result.

## Conclusion

With the current data, we concluded that the newly constructed and readily available nomogram model was able to predict the OS of patients receiving R0 resection and that it could be used to guide the management of p-TACE. However, the model needs additional external validation.

## Supporting information

S1 Fig The cut-off value of the neutrophil to lymphocyte ratio. (TIFF)

S1 Checklist STROBE statement—checklist of items that should be included in reports of observational studies. (DOC)
