# Supplementary figures and images for: A simplified model for prophylactic transarterial chemoembolization after resection for patients with hepatocellular carcinoma
Source: PLoS One. 2022 Oct 31;17(10):e0276627. doi: 10.1371/journal.pone.0276627 (PMC9621457; doi:10.1371/journal.pone.0276627)

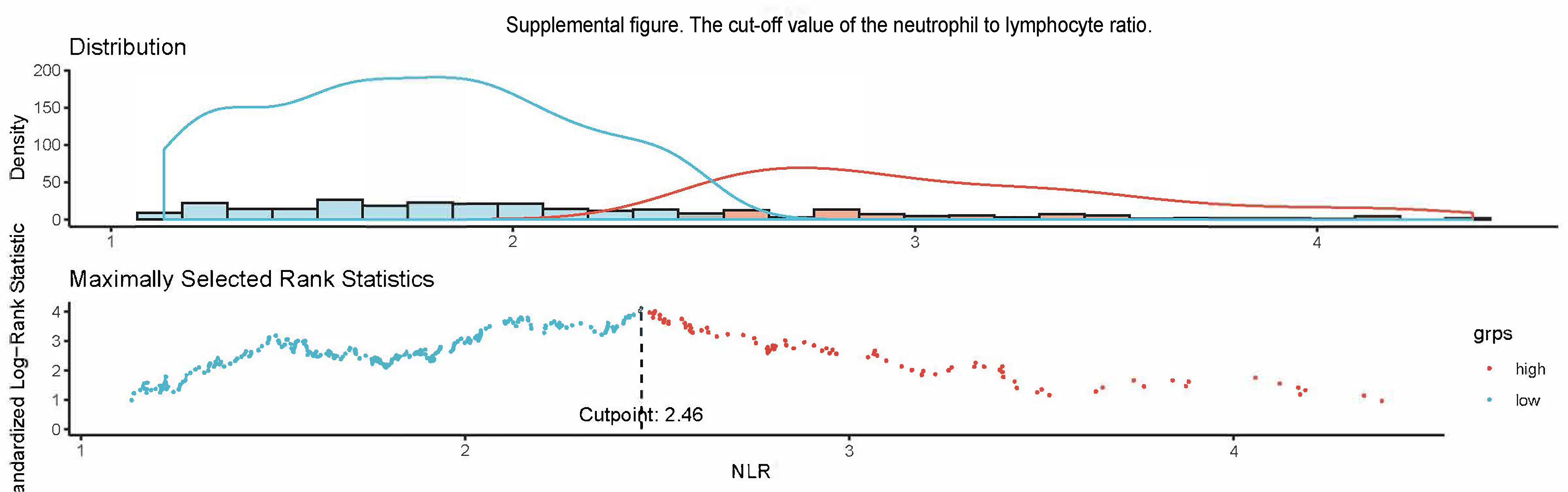

Supplement: S1 Fig — (TIFF) [file pone.0276627.s001.tiff]
